# *We other narcissists:* self-love in Freud and culture

**DOI:** 10.1080/0950236X.2020.1839956

**Published:** 2020-11-09

**Authors:** William Rees

**Affiliations:** English, UCL, London, UK

**Keywords:** Freud, psychoanalysis, narcissism, Laplanche, Levinas

## Abstract

I examine the paradoxical place of narcissism in contemporary culture, and within the work of Freud. Paying close attention to the repeated moments of equivocation and contradiction within Freud’s descriptions of primary and secondary narcissism, I draw on the work of Jean Laplanche, who suggests that the ambiguities in Freud’s texts often mirror ambiguities within the constitution of the ego. I argue that we should read Freud’s inability to rigorously distinguish self from other in his explications of self-love not – or not only – as a failure on his part, but also as a trace of an alterity at the heart of identity. It is the very ‘failure’ of Freud’s concept of narcissism that leaves it open to the other and makes it remain a vital concept today, when the word narcissism has been reduced to an impoverished notion of self-obsession. In closing I suggest that, with his knotted and never fully coherent concept of narcissism, Freud provides us with a way of thinking about human relationships outside of the binaries of selfless v selfish love that so commonly constrain our popular and theoretical ideas about love.

To a well-educated layman … things that have to do with love are incommensurable with everything else; they are, as it were, written on a special page on which no other writing is tolerated.

– Sigmund Freud, ‘Observations on Transference Love’^1^

[*Pause. He shrugs his shoulders, peers again at the ledger, reads.*] Farewell to – [*he turns page*] – love.

– Samuel Beckett, ‘Krapp’s Last Tape’^2^

## 1. I know you are, but what am I?

When I confessed, a few years ago, that I couldn’t truly say what the word *narcissism* meant, a friend suggested, ‘It depends which newspaper you read’. This was in 2015, when it still seemed feasible that Donald Trump would amount only to an inscrutable notation in the margins of history, and each day numerous articles appeared in newspapers across the political spectrum claiming to contain decisive evidence that Trump or Obama ‘is a narcissist’. These were joined by a steady stream of different articles, often in the form of lists and usually in the lifestyle or business supplements, which encouraged readers to view their personal and professional relationships as perpetually threatened by a legion of pathologically selfish mothers, bosses, and boyfriends, and to get ahead by learning how to ‘manage’ them. Meanwhile in yet another section of the newspaper, op-eds regularly informed readers (who nevertheless still formed an implied non-narcissistic ‘we’) that, in an age of selfies, auto-fiction, and fervid pop-psychologizing, more or less everyone, now, had become a narcissist.

Of course none of this is especially interesting. It has long been commonplace to refer to someone, whether casually or caustically, as a narcissist. More interesting, however, is how one seems to reveal so much about *oneself* – about one’s desires and fears – when using this word putatively to describe the personality of someone else. After all, the person whom I label a narcissist is typically someone who has refused to give me something (respect, an acceptable tax regime, unconditional love) that I both warrant and want, and so my diagnosis is motivated, at least in part, by my own frustrated desires. What interests me here is not the rightness, wrongness, or even the legitimacy of such lay diagnoses, but rather the element of *self*-revelation – as if whenever we use the word narcissist we are never quite sure whom we’re talking about.

Freud used the word *narcissism* to refer to a state of perfect self-absorption, an early stage of infantile development during which the other does not (yet) exist. Freud was not the first person to use the term, but without his intervention it is unlikely that narcissism would have fallen into the common purse. In his 1914 essay ‘On Narcissism: An Introduction’, he prepared the concept for mass use, assigning it not only to a coterie of perverts (as had the psychiatrists and sexologists before him) but to every member of the species (SE 14, p. 74). The infant’s first love object, suggests Freud, is her- or himself, and this inaugurating act of self-love serves to constitute the ego by way of libidinal fiat. Freud therefore placed narcissism in every person’s infancy. Yet for Freud narcissism does not remain buried in some deep past, but is continually ‘born again, … transformed into object-love’ (SE 14, p. 91); and his essay is, in part, a catalogue of the ways in which, in adulthood, narcissism slyly burlesques its supposed opposites. Erotic and parental love, he suggests, may ultimately be only dressed-up forms of self-love. One of the tasks of psychoanalysis is to unveil this disguised narcissism: to teach the patient that it may have been his own reflection he was contemplating in his lover’s eyes.

However, Adam Phillips suggests that the analyst’s debunking critical gesture is always made from shaky ground. In his elegant essay ‘Narcissism, For and Against’, he argues that psychoanalysis always has trouble distinguishing itself from the narcissism about which it theorises. Theories of narcissism are always (also) negative ideals of what constitutes a good life (say, a life that involves a measure of selfless love); and so these theories always reveal a good deal – and if, like Phillips, we are psychoanalysts, then probably a good deal more than we would like – about our moral values, our (unacknowledged) universal prescriptions for life. In other words, the only way that psychoanalysis can define narcissism is by being grandiosely omniscient about how a person ought to be and to live; and so: ‘no one is more narcissistic than the enemies of narcissism’.^3^

Of course the narcissist is always someone else, yet there does seem to be something ‘catching’ in its logic; it’s as though as soon as the word *narcissist* is invoked, a dialectic comes into play, and one can never be quite sure which side of it one stands on. In recent years, however, it has been far from only psychoanalysts who have used the word, and so perhaps a more pressing question than the one that Phillips asks is: what motivates our ‘wild’ theorising about narcissism? What is gained, emotionally or rhetorically, when we move from a moral to a medical register; when we call some ne’er-do-well partner or president not ‘bad’, but ‘mad’ – a narcissist? We can find a clue by looking to the enormous self-help literature that has accrued around narcissistic personality disorder. One of the axioms of that genre is that narcissists simply ‘cannot’ engage with other people’s points of view; that, fundamentally blinkered, they are ‘incapable of change’. In this way, a narcissism diagnosis rigorously separates self from other, and, whether expressed as pity or as censure, puts the onus for some failure of relation or communication squarely on the (now pathological) other. The person who has been so diagnosed has effectively been escorted from the conjectural arena: a narcissist, she cannot be otherwise than what she is, and it would be misguided or maddening to try to win her over in argument. So it will come as no surprise that the standard advice for how to ‘survive’, ‘leave’, ‘defeat’, or, more ambiguously, ‘deal with’ such a person is invariably that one should cease to entertain *their* point of view: *disengage*.

Perhaps with narcissists, then, it takes one to know one, and it is a common enough view that the self-help genre into which these books fall is itself symptomizing, aggravating, or possibly causing widespread self-obsession. This was the view taken by Christopher Lasch in *The Culture of Narcissism* (1979), a book whose own wild success owed something to a culture desperate to hear about itself, even – or especially – the very worst. In recent years, however, something does seem to have changed in the popular literature about narcissism. Where plenty have interpreted the world, and where Lasch perhaps still wanted to change it, the aim in this more recent literature has typically been restricted to personal survival. Enumerating the ‘warning signs’ and ‘red flags’ of narcissistic personalities, countless books and magazine articles today encourage readers to be carefully calculating in their social interactions, to vigorously protect the boundaries of their selves: ‘In the pages ahead, you’ll learn to spot Extreme Narcissists among your friends, family, and co-workers’, promises one popular book whose subtitle is ‘Defending Yourself Against Extreme Narcissists in an All-About-Me Age’.^4^ The world which these books describe resembles that of the modern zombie narrative: a desolate wasteland populated by pathological others with whom an ethical face-to-face is impossible. Yet this also means that it is one in which all the old scruples are relaxed which would impede me pursuing my own ends. Indeed, if the tone of these books is fatalistic then it is hardly depressive, often giving license to a dizzying, exhilarating nihilism. And so if for nothing else, then the bestselling *Becoming the Narcissist’s Nightmare: How to Devalue and Discard the Narcissist While Supplying Yourself* – published ten days after Trump won the Republican candidacy – is a book that can surely be celebrated for the way it makes explicit a worldview that is becoming far more widespread: if you can’t beat them … 

*

In what follows I want to return to Freud’s struggles to elaborate a theory of narcissism, roughly between the time of the Leonardo essay and up to the emergence of the second topography. As we will see, here, as well, there is a complicated relation between self and other, which manifests as an otherness that can never quite be banished from Freud’s attempts to define narcissism as a state of monadic self-isolation. Freud often seems at pains to argue that love of others disguises a foundational love of self; and yet each time he attempts to elaborate it, that love of self, too, seems curiously other-oriented. Drawing out, through close reading, these entanglements of self and other in Freud’s theories of narcissism, I want to suggest that we might see them not, or not only, as failures on his part, but also as suggestive traces of an alterity at the heart of identity. And so if my method entails paying the kind of close attention to Freud’s essays that is typically reserved for literary texts, then this is motivated by a belief that the ambiguities in Freud’s texts might reveal ambiguities in the objects they explicate. Freud fails to outline a fully coherent picture of narcissism – to distinguish, once and for all, ‘us’ from ‘them’, self from other, non-narcissists from narcissists. However, it is in this very failure that we can find his curious success. Above all, I wish to suggest that, if we can free it from its contemporary reductions, the concept of narcissism remains a valuable tool that can can allow us to move beyond the facile binaries of selfish *v* selfless love that still abide in many of our popular and theoretical conceptions of human relationships.

## 2. Getting over oneself

The term *narcissism* was coined in 1899 by Paul Näcke, who used it to describe a subject who treats himself like a sexual object. (A year earlier, Havelock Ellis had described a similar phenomenon, using the term ‘Narcissus-like’.) Freud begins his 1914 ‘On Narcissism: An Introduction’ by dismissing Näcke’s definition, suggesting that narcissism is in fact something far farther-reaching, not merely ‘a perversion, but the libidinal complement to the egoism of the instinct of self-preservation, a measure of which may justifiably be attributed to every living creature’ (SE 14, pp. 73–4). This ‘primary’ and normal narcissism which is Freud’s concern (though, as is his wont, he approaches it indirectly through the ‘distortions and magnifications’ of its perverse and pathological secondary reiterations) is a moment in ‘the regular course of human sexual development … , an original libidinal cathexis of the ego’, which serves to constitute that ego out of the originary formless chaos of the sexual drives (pp. 73–5). The infant, whose functional biological satisfactions become increasingly sexually charged, invests that nebulous surplus energy in its own nascent self. Taking itself as its first lover, the infant finds itself besotted by itself, ‘possessed of every perfection that is of value’ (p. 94). Eventually, however, when a reality grown impatient will wait no longer, the infant will be forced to open up, step outside, and so surrender this ‘blissful state of mind’ (p. 89).

Yet narcissism ‘fundamentally persists’ beyond this initial, isolated state (p. 75). That first libidinal endowment establishes the ego as a kind of central reserve bank from which future payments to others will be drawn – albeit always in cash. Or to use one of Freud’s own metaphors, the narcissistic ego, as he writes in ‘Mourning and Melancholia’, is

the great reservoir from which the object-cathexes are sent out and into which they are withdrawn once more; the narcissistic libidinal cathexis of the ego is the original state of things, realized in earliest childhood, and is merely covered by the later extrusions of libido, but in essentials persists behind them. (SE 7, p. 218)

In her essay ‘American Narcissism’, Louise Glück writes that Narcissus ‘is all psychology, no narrative … , a static image’.^5^ Narcissus, she suggests, exists for the modern imagination not so much as narrative, but as the single fixed image of fatal, fascinated self-regard. With his doctrine of primary narcissism, Freud would appear to have given us another static image of stasis. Freud makes self-love the inaugural act of human subjectivity, what, in a later summary of the narcissism essay, he calls an ‘original, primal distribution of libido in human beings’; at ‘the beginning of the development of the individual all his libido is tied to himself’ (SE 17, p. 138). Itself a moment without history, narcissism is the moment which founds the subject’s history: the instalment to the throne of His Majesty the Ego. And while, later, the subject will, if the ordinary life-story runs its course, experiment with the other kinds of loving, these forays in object-relations will always remain tentative, precarious modifications of that original and abiding self-love.

If, as this accounts suggests, the subject begins closed unto itself, then it is unclear how – or, indeed, whether – it could ever be prized open. In the latter parts of the essay, Freud finds narcissism hiding in every putative object-relation. In some famous, strangely touching lines he goes so far as to say, ‘Parental love, which is so moving and at bottom so childish, is nothing but the parents’ narcissism born again, which, transformed into object-love, unmistakably reveals its former nature’ (p. 91). In the West, the culmination of our redemption stories, whether theological or secular, has typically involved the transcendence of self through some form of ‘selfless’ love – whether of God or Neighbour, of Nation, Son, or Beloved. However, Freud brings the bad news that one always ends where one started. One’s love for the Other always disguises a love of oneself; one never truly leaves the mirror.

Defining the subject’s history in this way involves making a drastic, and constricting, claim about its future. It is therefore worth recalling that Freud’s *use* of the term narcissism has a history, making its entry into his work some years before its formal 1914 ‘Introduction’. Given the importance it will quickly accrue – in the work of Freud and in the popular imagination – narcissism’s entry is a little inconspicuous, appearing first of all in a footnote buried in the second edition of *Three Essays On The Theory Of Sexuality* (1910). Here Freud invokes Narcissus to explain the genesis of homosexuality (‘inversion’), drawing upon the cultural commonplaces that homosexuality has some important connection with vanity and with the too-tender ministrations of the mother. In infancy, we are told, inverts become obsessed with an over-affectionate mother (or mother-surrogate); having overcome that obsession, they later identify with her, taking or mistaking themselves for their preferred sexual object. ‘That is to say, [inverts] proceed from a narcissistic basis, and look for a young man who resembles themselves and whom *they* may love as their mother loved *them*’ (SE 7, p. 145).

The argument is further elaborated a few months later when Freud publishes ‘Leonardo da Vinci and a Memory of his Childhood’ (1910). In this essay we learn about the insidious forces bearing upon the nascent narcissist: effete and absent fathers, masculine mothers, ‘feminine influence’. Freud now associates narcissism with a reversion to autoerotism: ‘What he has in fact done is to slip back to auto-erotism: for the boys whom he now loves as he grows up are after all only substitutive figures and revivals of himself in childhood’ (SE 11, p. 100). But autoerotism here seems to have taken on a very different meaning to the one given it five years previously in the second of the *Three Essays* (1905), where it referred to the free-floating, objectless sexuality of early infancy. One could call this revised concept a *secondary* autoerotism, perhaps, though I’m not convinced that this would be quite right. Because what’s curious about Freud’s gender-bending psychodrama in the Leonardo essay is that the narcissist’s self-love is in fact a disguised way of remaining faithful to the (*m)other*: ‘While he seems to pursue boys and to be their lover, he is in reality running away from the other women, who might cause him to be unfaithful’. This ‘autoerotism’, far from excluding all others, in fact involves a kind of three-way: subject, mother, and same-sex love object. And so if, in these essays, narcissism is modelled on the self, then it is underwritten by the presence of others. Not quite autoerotism and not quite object love, narcissism is – what exactly?

In his 1911 essay on the paranoid German judge Daniel Paul Schreber, Freud returns to and drastically rethinks the concept of narcissism. Once again, he is concerned with the aetiology of homosexuality; however, no longer simply a type of object choice, narcissism is now elevated to the status of a developmental stage, one related to yet distinct from autoerotism. ‘Recent investigations’, says Freud, citing his Leonardo essay, ‘have directed our attention to a stage in the development of the libido which it passes through on the way from auto-erotism to object-love’ (SE 12, p. 60). Narcissism is a ‘half-way phase between auto-erotism and object-love’ (p. 61). No longer the perverse regression to infantile autoerotism, narcissism now names the healthy consolidation of the infant’s chaotic sexual drives, their being brought to bear on his first love object: himself. The infant ‘begins by taking himself, his own body, as his love-object, and only subsequently proceeds from this to the choice of some person other than himself as his object’ (pp. 60–1). In this essay narcissism, no longer or not only perverse, has become a moment in the ordinary ascent from infancy to mature, normative subjectivity.

However, if narcissism is a rung on this ladder, then it is also potentially a loose one. ‘[I]t appears that many people linger unusually long in this condition’, writes Freud, ‘and that many of its features are carried over by them into the later stages of development’ (61). Quite how many people linger in this condition, and what happens to them (presumably not all of them turn out like Judge Schreber), Freud, for now, does not say. But if narcissism is a normal developmental stage, then it nevertheless remains a snare, a temptation that threatens to structure the subject’s later erotic life. One’s first love, Freud would seem to imply, might be the hardest to get over.

## 3. An open-and-shut case?

All of which is to say that Freud’s famous 1914 ‘Introduction’ in fact rearticulates, while attempting to systematize, insights that had recurred throughout his work for almost half a decade. And that the narcissism it explicates is perhaps not quite as ‘closed’ as it first appears; the theory inherits some holes. By introducing several important conceptual distinctions – between ego- and object-libido, primary and secondary narcissism – Freud’s essay answers some pressing questions posed by his so-far fragmentary and occasional offerings on the topic. But it poses many more. Despite the apparent simplicity of its central idea, ‘On Narcissism’ must surely rank among Freud’s most confounding, and confounded, essays – as though the clarity it seeks to impose through its technical distinctions only served to emphasise the conceptual chaos underlying it.

Rather than providing a comprehensive gloss, in what follows I want to draw attention to two moments of ambiguity in the essay, which have their roots in those earlier accounts. The first moment occurs when Freud attempts to clarify the distinction between narcissism and autoerotism; this, he candidly admits, is one of the

questions which lead us to the heart of the difficulties of our subject. In the first place, what is the relation of the narcissism of which we are now speaking to auto-erotism, which we have described as an early state of the libido? (p. 76)

We are, he continues, ‘bound to suppose that a unity comparable to the ego cannot exist in the individual from the start; the ego has to be developed. The auto-erotic instincts, however, are there from the very first’ (pp. 76–7). At this point a footnote in the standard edition sends us back to the second of the *Three Essays*; yet if we follow that reference we in fact learn that Freud must have significantly, if silently, revised his opinion on autoerotism between the 1910 edition of the *Three Essays* and the present text. Because in 1905/1910 autoerotism was precisely *not* there ‘from the very first’. In the *Three Essays* autoerotism (another word that Freud borrows from Havelock Ellis) denotes sexuality ‘not directed towards other people, but  … the subject’s own body’ (SE 7, p. 181). Freud finds the ur-scene of autoerotism in the infant sucking its thumb, which unsurprisingly is modelled on the memory of the breast. He adds, ‘The satisfaction of the erotogenic zone [in this case the mouth] is associated, in the first instance, with the satisfaction of the need for nourishment [i.e. by the mother’s nursing]’ (pp. 181–2). In other words, autoerotism arises as a side effect of maternal care, and only later can it be separated from the activities of mothering. Referring only to the self, autoerotism nonetheless depends on the presence of the other. For Freud, at least in the *Three Essays*, without the presence of the other there can be no sexuality.^6^

‘[T]here must’, Freud goes on to say in ‘On Narcissism’, ‘be something added to auto-erotism – a new psychical action – in order to bring about narcissism’. This suggests that narcissism is not as original or as ‘static’ as it first appears; it is the result of an ‘action’, a fencing off, a cathexis which closes something that had previously been open. Freud, as we have seen, nevertheless does posit a state of sexualised isolation – a biological monadism that exists within an even deeper past (the ‘autoerotism’ of the *Three Essays*). And yet when we try to follow him there it seems to recede on approach – for autoerotism, too, it turns out, requires the presence of the other, and so, far from being a state of isolation, in fact implies a social relation. Thus: Freud’s account of narcissism and ego-formation appears to require, but to be unable to locate, a state of isolated monadic enclosure.

The second moment I’d like to draw out is Freud’s attempt to decide whether primary narcissism should be understood as a stage or as a structure. Is narcissism a (surmounted) moment in each adult’s past, or the abiding structure of libido? We saw that in the Schreber essay narcissism was proposed as an infantile developmental stage. However, if narcissism is indeed a stage in the subject’s prehistory, then it is unclear how the subject could emerge from it. As Laplanche and Pontalis point out in *The Language of Psychoanalysis*, the notion of narcissism-as-stage looks vulnerable to the trap of metaphysical idealism, the ‘problem of beginnings’. How, they ask, is the ‘monad shut in upon itself’ ever supposed to gain entry into the world of things? In addition to this philosophical problem, they write, ‘there is a danger of running counter to experience by asserting that the newborn baby is without any perceptual outlet on to the external world’.^7^

For Laplanche and Pontalis, ‘On Narcissism’ is the moment where Freud was ‘brought … to define narcissism *structurally*: instead of appearing as a developmental stage, narcissism now emerges as a damming up of the libido which no object-cathexis can completely overcome’.^8^ But this is one of those rare moments where Freud’s position is less clear-cut than that of his French readers. In fact, Freud’s former account has neither been supplemented nor supplanted: narcissism now appears suspended between stage and structure. Because at the beginning of the paper, Freud rehearses his previous theory that narcissism is a developmental *stage* – doing so, however, as though this were a piece of evidence for the economic *structure* of the narcissistic libido (SE 14, pp. 74–5). If in the Schreber essay narcissism is a stage that ‘many people linger unusually long in’, Freud now appears to apply that diagnosis universally. In answer to the question of whether narcissism is a stage or a structure, the text would seem to propose, both; but to do so in such a way as to make it difficult to maintain either.

## 4. Between two points of view

Given these ambiguities in ‘On Narcissism’, it is not surprising that many psychoanalytic critics have rejected the concept it proffers out of hand. Those within the object relations tradition have suggested that, from birth, the infant begins libidinally cathecting external objects. This would mean that narcissism is always secondary. This is Michael Balint’s claim in *Primary Love and Pyscho-analytic Technique* (1952), in which he rejects Freud’s primary narcissism and replaces it with ‘primary object-love’. Balint draws our attention to and capitalises on the chaos within Freud’s text – the way it ‘oscillates between two points of view’.^9^ He criticises Freud’s concept for its purported negativity, emptiness of content, and wholly formal, inferential nature, and he concludes that primary narcissism is ‘full of meaning and yet very poor’, based, as it is, on the erroneous belief that ‘the logically simple is necessarily the chronologically earlier’.^10^ ‘Primary narcissism’, he writes, ‘bars the assumption of any relation to external objects’, which indeed does make it sound quite absurd. ‘Already Freud has emphasised that absolute narcissism in itself is impossible because a living being in this state is not viable’.^11^ Primary narcissism cannot even be sustained within the texts which argue for it; on Balint’s reading, ‘On Narcissism’ becomes a neat demonstration of the impossibility of its own central thesis.

However, in his inversion of what he takes to be Freud’s crudely self-invested self, Balint remains caught within the logic of that binary: replacing one point of view for another. Only by attending carefully to the ambiguities of Freud’s essay might we avoid the haphazard recycling of such binaries. These ambiguities are at least in part the mark of a transitionary, nodal work, one which tries and fails to carry the load of too much theoretical baggage. (While attempting to introduce a major new concept, it also forms the occasion for Freud to engage in polemics against Jung and Adler, who in different ways had deviated from libido theory.) Meanwhile we learn from Freud’s correspondence, quoted in Strachey’s introduction in the Standard Edition, that the essay was written in haste; that Freud considered it to have had ‘a difficult labour’, and to bear ‘all the marks of a corresponding deformation’ (p. 70). Despite these quirks of composition, it would, I think, be wrong to reduce the paper’s ambiguities to accidental properties: they are (if only in spite of themselves) revealing.

In the introduction to his 1970 *Life and Death in Psychoanalysis*, Jean Laplanche makes the following claim: ‘The contradictions in Freud’s thought and the contradictions in his object are, in the final analysis, inseparable’.^12^ For Laplanche this insight seems to be both the prize and the rule of the game: it both derives from and guides his reading. While wishing to remain agnostic as to its truth, I want to take this as a kind of hermeneutic heuristic, a regulative idea for a reading of Freud. Doing so will allow us to ask whether those intrusions of alterity which Freud is at pains, and yet unable, to suppress in his account of narcissism, might be taken to reveal something about ‘the object’. In other words: if others keep intruding in Freud’s account of selfhood and narcissism, then might this in fact indicate something about that self and its narcissism?

We find the clearest statement of the stakes of Laplanche’s project in his popular synoptic essay, ‘The Unfinished Copernican Revolution’. At the end of his 18th Introductory Lecture of 1916–17 Freud famously, and grandiosely, positioned himself as the latest in a line of thinkers – Copernicus and Darwin were the other two – who had committed devastating ‘blows’ against human narcissism, decentring man in relation to the universe, then the species, and now, with the discovery of the unconscious, in relation to himself. As his title suggests, Laplanche doesn’t so much wish to dispute the existence of this radical, ‘Copernican’ force in Freud’s thought as to suggest that its work remains incomplete. This force, he says, is always acted upon by a counterforce of narcissistic *re­-*centring, with the two often being operative within the very same texts: ‘if Freud is his own Copernicus, he is also his own Ptolemy’.^13^ However – and this is crucial – this narcissistic re-centring is not merely a failure on Freud’s part, because it in fact mirrors opposing tendencies *within the human ego*:

in Freud the theoretician, the going-astray is accompanied by a sort of connivance with the object; in other words, a covering-up of truth inherent in the very object to which thought conforms. The closing-in-on-itself of the Freudian psychical system, its *monadological* character … would be radically linked to the closing-in-on-itself of the human being in the very process of its constitution.^14^

Where Balint finds only incoherence, for Laplanche, ‘Freud’s endless Ptolemaic relapses’ mirror the oscillations of the ego, which, never coinciding with itself, nevertheless constantly seeks to become the centre of its own existence (a revolution which always remains ‘unfinished’).

*

In *Life and Death in Psychoanalysis,* Laplanche asks: ‘by what necessity [do] both narcissism and the ego pass themselves off to us, mythically, as “primal”’?^15^ Indeed, it is striking that each time Freud speaks about narcissism, he does so by employing mythic devices. Most obviously there is the image of Narcissus himself, the beautiful child doting unto death upon his own reflection. Then there is the prelapsarian intrauterine state, which Freud calls ‘blissful’ and from which one is always wrested ‘too early’. In his 1911 ‘Formulations on the Two Principles of Mental Functioning’, Freud finds an image of narcissistic enclosure in the bird’s egg with its food supply encased within its shell. In this essay, Freud provides a genealogy of the reality principle, which develops gradually, over time, due to the progressive disappointment of the originary pleasure principle. He describes the originary stasis – or ‘state of psychic rest’ – of a self-enclosed, self-sufficient infant whose drives are satisfied ‘by means of hallucination’ (SE 11, p. 219). In the earliest stages of its development, the infant need not even ask and it is given: no sooner has it become aware of its hunger than satisfaction floods in to erase that discomfiting sensation.^16^ Freud explains that with the gradual awakening of the reality principle, which is occasioned by the ‘persistent absence of satisfaction’, the hermetic infant, now disabused of its imaginary omnipotence, slowly makes its way out into the world of things. He explains: ‘A neat example of a psychical system shut off from the stimuli of the external world, and able to satisfy even its nutritional requirements autistically, … is afforded by a bird’s egg with its food supply enclosed in its shell’ (SE 11, p. 220n). The foetus in its shell would be akin to the earliest infantile state, in which the infant, closed unto itself, has its drives satisfied without forming any psychical representation of the reality that satisfies them.

It is not difficult to spot the problem within the analogy. The foetus’s self-sufficiency is only illusory, relying as it does upon the care of a mother that protects and warms it – thus instantly problematising this simple analogy. The same of course is true of the infant, whose satisfactions rely upon the mother’s watchful ministrations. Freud himself notes this with the very next line, admitting that such ‘an organization … could not maintain itself alive for the shortest time’ (p. 220n). This moves Balint to write,

Already Freud has emphasised that absolute narcissism in itself is impossible because a living being in this state is not viable. Ever since, following his example, we quote in this connection the nursing environment … This primary state is only possible in the form of the mother–child unit.^17^

For Balint this is the moment where, against his intentions yet by his own hand, Freud’s account of primary narcissism slips into primary love. However, when Freud has made the above admission, he then adds that this ‘*fiction* … [is] justified when one considers that the infant – *provided one includes with it the care it receives from its mother –* does *almost* realize a psychical system of this kind’ (p. 220n; my emphasis). These are strange lines, but I’m not convinced that we can read them, as per Balint, as an admission of a poorly constructed analogy elucidating a poorly constructed theory.

In fact Freud seems to be pre-empting Balint’s attack, which suggests that he does not see it as a threat. Julie Walsh writes that Freud has ‘anticipated Balint’s basic criticism’ and that for Freud ‘the presence and the care of the mother (or nursing environment) is taken as read’.^18^ Indeed, with these ‘admissions’, Freud’s point is surely that this state of narcissistic self-enclosure is an infantile illusion, a ‘fiction’ that requires the presence of a loving other in order for it to be sustained. Josh Cohen writes that ‘it is the very fact of infantile helplessness which conditions its illusions of autonomous self-enclosure. The objectless state is the paradoxical effect of the maternal object’s care’.^19^ If this is right, then Freud is not making the patently absurd claim that the narcissistic infant exists without a relation to its environment, or to others; in fact Freud’s account *requires* that the infant be in a relation to its environment and to others. That relation, though, is one that is, for a time, disavowed. The threatening environment forces the infant into illusory self-enclosure; and maternal love allows the illusion to persist, for a time; allows the psychical system laid out above to be ‘almost realised’, before it is eventually and gradually opened up (a work which remains unfinished). The paradoxical conditions for narcissism, then, are that the infant be at the mercy of the world, and that there be a loving other to grant that mercy.

In his reading of Freud, Laplanche rejects the notion of narcissism represented by Freud’s metaphor of the egg, which he deems to be the genesis of everything that goes wrong in Freud’s theorising as it takes on an increasingly monadic dimension.^20^ But what Laplanche does not notice is that it is precisely because this image appears contradictory that a path is opened. The primary illusion of monadic self-enclosure requires a social relation. Perhaps this theory would never have found favour with Laplanche because it looks, at least on the face of it, as though the other only plays a negative role: it is, after all, through the other’s absence, not her presence, that the subject begins to be disabused of its illusory self-enclosure. But to be disabused of an illusion requires, first, that one believe it, and that requires that one be *seduced* by it. Laplanche famously assigns quite a different meaning to seduction, emphasising the traumatic, radically unassimilable nature of the other’s enigmatic ‘messages’: these messages become lodged in me, they cannot be processed, and thus is born the unconscious as an internal otherness, a ‘strangerness’ or ‘alien-ness’ inside me.^21^ Yet I wonder whether the model of narcissism proffered in ‘Two Principles’ might constitute a complement to Laplanche’s account of the parent–child relation as a seduction into subjecthood.

Recall Freud’s comment in ‘On Narcissism’ that ‘a unity comparable to the ego cannot exist in the individual from the start; the ego has to be developed … there must be something added to auto-erotism – a new psychical action – in order to bring about narcissism’ (SE 14, p. 77). If the other plays a part in the narcissistic formation of the ego, in the mythic illusion of self-enclosure – which will gradually diminish but which will ‘fundamentally persist’ and continue to determine psychical life – then that ego is split from the first, and its ‘unity’ is always a broken unity. Such a narcissism would remain precarious, provisional, and open – like object-love. Balint writes: ‘A narcissistic attitude should make one independent of the world. Experience teaches us, however, that … narcissistic peoples [*sic*] are almost paranoid-hypersensitive, irritable … The same is true of children’s behaviour from the very beginning’.^22^ But if the other is implicated in my narcissism, then we can see that this need not be true; founded in its opposite, love of self, like love of others, would be mobile, labile, and perpetually vulnerable. That the narcissistic ego is independent of the world is, on this account, simply the myth it tells about itself – one of the ruses by which, as it turns inside and out, it attempts to occupy the centre of its existence. Or in Laplanche’s terms, neither Ptolemy nor Copernicus has the last word. Or to put it yet another way, if Freud’s text, as Balint argues, ‘oscillates between two points of view’, then must we necessarily see that as a mistake?

## 5. A call from *inside*

At the beginning of Franz Kafka’s fable ‘The Burrow’, the narrator declares: ‘I have completed the construction of my burrow and it seems to be successful’.^23^ However, as the unnamed creature works to secure its enclosure from any possible intruders, it becomes increasingly unsettled as the sturdy walls of its burrow fail to keep out encroaching doubts:

the burrow does provide a considerable degree of security, but by no means enough, for is one ever free from anxieties inside it? These anxieties are different from ordinary ones, prouder, richer in content, often long repressed, but in their destructive effects they are perhaps much the same as the anxieties that existence in the outer world gives rise to.^24^

Eventually the creature hears a sound, ‘an almost inaudible whistling noise’, detectable ‘only to the ear of the householder’, but which, once heard, cannot be unheard. Alone unto itself and in complete narcissistic isolation, Kafka’s creature is nevertheless not at ease; instead it is haunted by an *internal* otherness, one that turns out to be more unsettling than anything in the outside world from which the hermetically sealed burrow had been intended as a means of escape. This internal otherness is typically the more or less terrifying discovery within the classic haunted house tale. Enervated by the abrasive alterity of urban life, the protagonist seeks to escape the city. He decamps to the country where he will not be disturbed, closes the doors and windows on the world – and then the walls begin to murmur. Those murmurings will invariably be horrifying, far more ‘other’ than the inane chatter of the city, and soon the protagonist longs, once again, for society, as if there and only there, surrounded by others, could he be truly himself. For in the cloistered intimacy of the *chez moi*, one finds a more frightful kind of otherness, what Laplanche calls an ‘inner “foreign body”, which now breaks out from within the subject’ – the ‘other in me’, the call that is coming from *inside* the house.^25^

A similar theme recurs within Freud’s texts. Each time he tries to elaborate an example of narcissism, the rigid binary he posits between self and other appears to break down – as if his descriptions of narcissism were haunted by an otherness they are quite unable to banish. For instance, in the 1914 essay, Freud writes that sleep is a daily accomplishment of total narcissism, calling it the ‘narcissistic withdrawal of the positions of libido on to the subject’s own self, or, more precisely, on to the single wish to sleep’ (p. 83). Sleepers retreat into themselves, ‘lay[ing] aside … their psychical acquisitions’ and undressing their minds like they undress their bodies (SE 14, p. 222). One by one I cast off my worldly investments, and when sleep comes the world slips away, and with it all that is other. However, if sleep is the apotheosis of narcissism, then it is also the moment at which I become least myself, lose my self, become other. Jean-Luc Nancy makes this point in *The Fall of Sleep*: ‘By falling asleep, I fall inside myself … I sleep and this *I* that sleeps can no more say it sleeps than it could say that it is dead’.^26^ For Nancy, these two moments – coming into myself, losing myself – are part of one and the same movement:

I now belong only to myself, having fallen into myself and mingled with that night where everything becomes indistinct to me but more than anything myself. I mean: everything becomes more than anything myself, everything is reabsorbed into me without allowing me to distinguish me from anything. But I also mean: more than anything, I myself become indistinct.

Nancy concludes: ‘So it is another who sleeps in my place’.^27^ Like the protagonist of the haunted house tale, in sleep one withdraws into complete psychic privacy, only to find waiting there another kind of otherness.

Meanwhile consider what, for Freud, became the archetype of narcissism: the vain woman. ‘Strictly speaking, it is only themselves that such women love with an intensity comparable to that of the man’s love for them. Nor does their need lie in the direction of loving, but of being loved’ (SE 14, p. 89). Freud’s aetiology is crude, yet illuminating: such women ‘develop a certain self-contentment which compensates them for the social restrictions that are imposed upon them in their choice of object’. This narcissistic state, therefore, refers not simply to the self, but to an entire social system. More notable still, however, is where Freud writes:

The importance of this type of woman for the erotic life of mankind is to be rated very high. Such women have the greatest fascination for men … because of a combination of interesting psychological factors. For it seems very evident that another person’s narcissism has a great attraction for those who have renounced part of their own narcissism and are in search of object-love. (p. 89)

In this scene, the man is (fatally?) fascinated by the narcissistic woman, in whom he finds the mirror image of his own (‘renounced’) narcissism. Is the narcissism Freud diagnoses in the woman, then, not (also) the fantasy of the fascinated male who sees in her an opportunity for blissful self-renunciation? It is not the point Freud wishes to make, and yet neither does it require a big leap to suggest, that narcissism, here, is a matter of projection and introjection: a *shared* fantasy; a role that is carved out, negotiated, and adopted *between two*.

Finally, consider what, in ‘On Narcissism’, Freud calls ‘the strongest of the reasons which have led us to adopt the hypothesis of narcissism’: the narcissistic object choice of ‘perverts and homosexuals’ (p. 88). We learned in the Leonardo essay that narcissistic object choice is an effect of an identification with the other, reiterating the fussy mother’s overvaluation. In ‘On Narcissism’, this structure appears to quietly become a generalised theory of *all* narcissism: ‘Parental love, which is so moving and at bottom so childish, is nothing but the parents’ narcissism born again, which, transformed into object-love, unmistakably reveals its former nature’ (p. 91). As Laplanche writes, this ‘sends us indefinitely from infantile narcissism to infantile narcissism, those “narcissistic states” that are alleged to be closed upon themselves, being observed from the only observable situation: the narcissistic object-choice or *relation* of parents to child’. And so, he writes, ‘One need only go a bit farther in the direction indicated by Freud’ to see the ‘megalomanic illusions of the child’ as introjections of an ‘inverted form of parental omnipotence’.^28^ On the standard interpretation of Freud, love of the other emanates from and refers back to a primary love of self, thus making it ever available to the reductive critical gesture in which every ‘other’ can be unmasked to reveal, once again, the beloved self. However, we might suggest that this is only half the picture. Because each time Freud attempts to elaborate an account, or even an example, of narcissism, the reverse also appears to be true: ‘I’ turns out to be another. In this way, Freud’s texts quietly suggest that narcissism exists only in relation: ‘primary’ self-love relies upon, and conceals, a relationship with the other. My narcissism is never wholly ‘mine’.^29^

## 6. A happy ending?

If we were to say that the notion of primary narcissism has been called ‘nebulous, scarcely imaginable’, then one might reasonably presume that the attack comes from Balint or Klein or one of Freud’s many other antagonists. In fact the words belong to Freud himself (SE 14, p. 77). Balint criticises Freud’s theory of narcissism for being ‘not an observable fact but a hypothesis based on theoretical extrapolation’.^30^ Again, however, Freud had already said as much in the narcissism essay itself, where he writes that the ‘primary narcissism of children which we have *assumed* and which forms one of the postulates of our theories of the libido, is less easy to grasp by direct observation than to *confirm by inference from elsewhere*’ (SE 14, p. 90; my emphasis). For Freud, primary narcissism is an ‘assumption’, a hypothesis based not on clinical observation of children, but – belatedly – on its manifestations in the psychical lives of adults.^31^

To understand this, we need to look at the comments that Freud makes when he is differentiating the psychoanalytic search for origins from that of speculative metaphysics: whereas the latter seeks a sharply defined conceptual foundation on which a system will subsequently be erected, psychoanalysis, like the empirical science which Freud (rather wishfully) believed it to be, starts from experience and then contents itself with

basic concepts, which it hopes to apprehend more clearly in the course of its development, or which it is even prepared to replace by others. For these ideas are not the foundation of science, upon which everything rests: that foundation is observation alone. *They are not the bottom but the top of the whole structure*. (p. 77; my emphasis)

Freud is not, as Balint would have it, the philosophical novice supposing that ‘the logically simple is necessarily the chronologically earlier’. For Freud, primary narcissism is a hypothesis about the genesis of the ego, which is neither derived from direct observation, nor postulated as a first principle, but which is posited – tentatively – based on the observation of psychic lives of adults. This helps us understand the ambiguity in ‘On Narcissism’ as to whether narcissism is a stage or a structure. Freud posits narcissism as the structure of the subject’s libidinal life, the origins of which he speculatively locates in an only imaginatively accessible stage in the infant’s prehistory. In this sense, this account of ontogenesis ought to be read alongside the account of phylogenesis proffered, the previous year, in *Totem and Taboo* (1913) – that is, as a creation *myth*. Or better still, in Laplanche’s words, as an attempt to understand the way that ‘narcissism and the ego pass themselves off to us, mythically, as “primal”’.^32^

*

The irony above is that it is Freud’s appeal to the standards of empirical science that marks the entry point of fiction into his thought. However, if Laplanche is correct, then there is ongoing ‘literary’ activity within the ego, in the sense that its narrative about itself is always a fiction. Forever trying to place itself at the centre of its own existence, the ego is a constant self-mythologizer. Yet perhaps this also dramatises the deeper tension between art and science that marks Freud’s work: the way that, as he famously observed about his case studies, everything he writes seems unavoidably to become literature. In the Leonardo essay, Freud worries that he may have ‘only written a psychoanalytic romance’, thereby raising the question of genre – a question which, in closing, I would like to reflect upon.

I have insisted throughout that one might best approach Freud’s texts by attending to their literary dimensions. In his recent book about the philosopher Emmanuel Levinas, Simon Critchley makes an analogous claim, arguing that *Totality and Infinity* (1961) is best read not as a work of philosophy, but as a drama. If Levinas has indeed written a drama, then what sort of drama is it? While this may come as a surprise to many readers of that book, according to Critchley it is a comedy – specifically a divine comedy.^33^ In his book’s final chapter, Critchley seeks to reclaim erotic love from the philosopher’s wastepaper basket, arguing that eros – and not non-concupiscent, ethical love – is the correct answer to the question that Levinas set himself: the question of how one might transcend the monotony of monadic identity. Critchley, however, has accepted what he takes to be Levinas’s terms of engagement. And so in order to exonerate erotic love, to make of it the culmination in his secular redemption narrative, first he has to cleanse it of self-investment by arguing that it is ‘the movement of decreation, the stripping away or negating of everything … that ties us to the self and world’.^34^ Yet this sounds like a fairly good description of what for psychoanalysis goes by another name: *masochism*. In his essay on that topic, Freud points out that ‘the true masochist always turns his cheek whenever he has a chance of receiving a blow’ (SE 19, p. 165). However, Freud is characteristically attuned to the possibility of autoerotism and self-aggrandisement where Critchley finds only self-abandon:

It is very tempting, in explaining this attitude, to leave the libido out of account and to confine oneself to assuming that in this case the destructive instinct has been turned inwards again and is now raging against the self; yet there must be some meaning in the fact that linguistic usage has not given up the connection between this norm of behaviour and erotism and calls these self-injurers masochists too (SE 19).

Indeed, the way that, for Critchley, the lover marshals the beloved into their scheme for transcendence might give us pause to question the purported purity of this ‘negation’: might Critchley’s lover not simply be the man who, for Freud, finds in the ‘fascinating’ narcissistic woman the mirror image of his own (renounced) narcissism? Earlier in his book, Critchley argues that philosophy has a symptomatic discomfort with drama, always seeking to resolve its ambiguities. And yet in his attempt to transcend the drama of human relationships, Critchley, too, feels the need to disambiguate love – thereby haphazardly repeating the philosopher’s perennial error.

I make this digression because I think that it shows precisely the kind of thinking that Freud, on the reading proposed here, allows us to be moving along from. In ‘On Narcissism’ Freud drolly writes that the way that ‘love’s feelings, however strong, are banished by bodily ailments, and suddenly replaced by complete indifference, is a theme which has been exploited by comic writers to an appropriate extent’ (SE 14, pp. 82–3). It is fitting that Freud should invoke the genre of comedy to describe the precariousness of love, its vulnerability to the bathetic incursion of a bodily self. Isn’t it this very precariousness which constitutes everything miraculous, and from a certain angle humorous, about love, a state which occasionally, and for a time, allows terrestrial embodied animals to entertain the notion that they have access to the eternal and unconditional? In place of the pomp and grandeur of Critchley’s ‘divine’ comedy, in which the self seeks its exquisite dissolution in masochistic love of the other, I propose then that Freud tells a story that is humbler, perhaps truer, and certainly funnier: a tragicomic romance; a notion of love in which the self persists, if only in spite of itself. But that very self is one that is split from the first, one in which the other is always already implicated. In this way, Freud allows us to think outside the binary of self and other which constrains the plurality of human relations and reduces them to a choice between selfish and selfless love – a choice which, needless to say, is really no choice at all. Or, indeed, a binary in which the other’s narcissism is never my affair, nor mine theirs.

## Notes

1 Sigmund Freud, *The Standard Edition of The Complete Psychological Works of Sigmund Freud*, Vol. 12, trans. and ed. James Strachey (London: Hogarth Press, 1958), p. 160. All subsequent references to Freud will be made in text, and will refer to the Standard Edition of his work in the format *SE vol, page number*.

2 Samuel Beckett, *The Collected Shorter Plays Beckett* (New York: Grove Press, 2010), p. 51.

3 Adam Phillips, *One Way and Another* (London: Penguin, 2013), p. 130.

4 Joseph Burgo, *The Narcissist You Know: Defending Yourself Against Extreme Narcissists in an All-About-Me Age* (New York: Simon & Schuster, 2016), p. 2.

5 Louise Glück, *American Originality: Essays on Poetry* (Farrar, Straus and Giroux, 2017), p. 7.

6 Curiously, it is *after* the narcissism paper that Freud renders this point even more explicit, adding several important sentences to the 1915 edition, such as ‘To begin with, sexual activity attaches itself to one of the functions serving the purpose of self-preservation and does not become independent of them until later’ (p. 182). It is not unusual for Freud to update his texts with new findings; however, this is entirely inconsistent with his claim in ‘On Narcissism’ that autoerotism is ‘there from the very first’.

7 Jean Laplanche and Jean-Bertrand Pontalis, *The Language of Psychoanalysis*, trans. Donald Nicholson-Smith (London: Karnac, 1988), p. 257.

8 Laplanche and Pontalis, *The Language of Psychoanalysis*, p. 216.

9 Michael Balint, *Primary Love and Psycho-Analytic Technique* (London: Karnac, 1985), p. 64.

10 Balint, *Primary Love and Psycho-Analytic Technique*, p. 103, p. 30.

11 Ibid., pp. 103–5.

12 Jean Laplanche, *Life and Death in Psychoanalysis*, trans. Jeffrey Mehlman (Baltimore: John Hopkins UP, 1976), p. 2.

13 Jean Laplanche, *Essays on Otherness*, ed. Jean Fletcher (Routledge, 2005), p. 61.

14 Laplanche, *Essays on Otherness*, p. 83.

15 Laplanche, *Life and Death in Psychoanalysis*, p. 72.

16 Though Freud does not call this primary biological enclosure *narcissism* – which at this point in his elaboration of it is still predominantly linked with homosexuality – his description of it is sufficiently close to the ‘original, primal’ state of narcissism (SE 17, p. 138) for us to presume that this is primary narcissism in nascent form

17 Balint, *Primary Love and Psycho-Analytic Technique*, p. 87.

18 Julie Walsh, *Narcissism and its Discontents* (London: Palgrave Macmillan, 2015), p. 27.

19 Josh Cohen, ‘“I-not-I”: Narcissism Beyond the One and the Other’, in Anastasios Gaitanidis with Polona Curk (eds), *Narcissism: A Critical Reader* (London: Karnac, 2007), p. 34.

20 Laplanche, *Life and Death in Psychoanalysis*, p. 72.

21 Jean Laplanche, *New Foundations for Psychoanalysis*, trans. David Macey (Oxford: Basil Blackwell, 1989), p. 89, p. 152.

22 Balint, *Primary Love and Psycho-Analytic Technique*, p. 105.

23 Franz Kafka, *The Complete Short Stories of Franz Kafka*, trans. Muir, Edwin and Muir, Willa (London: Vintage, 1999), p. 325.

24 Kafka, *The Complete Short Stories of Franz Kafka*, p. 339.

25 Jean Laplanche, *Essays on Otherness* (Routledge, 2005), p. 8.

26 Jean-Luc Nancy, *The Fall of Sleep*, trans. Charlotte Mandell (Fordham University Press, 2009), p. 7.

27 Ibid.

28 Laplanche, *Life and Death in Psychoanalysis*, pp. 78–9.

29 Although it would require a paper of its own to fully substantiate this claim, this interpretation of narcissism as requiring, and making space for, the other shares much in common with that of Jacques Derrida, in particular with what he came to call *autoimmunity*, the hyperbolic, self-defeating defensiveness that leaves one open to the other, and is therefore a paradoxical condition of hospitality, ethics, and democracy to come. For an excellent account of what Derrida can bring to a theory of narcissism, see: Pleshette DeArmitt, *The Right to Narcissism: A Case for an Im-Possible Self-Love* (New York: Fordham University Press, 2013).

30 Balint, *Primary Love and Psycho-Analytic Technique*, p. 87.

31 When Freud does promise to reinforce his views with ‘observations … on the mental life of children’, he does no such thing. Instead he draws a shaky analogy between the mental lives of children and those of half-heartedly imagined ‘primitive peoples’ (SE 14, p. 75).

32 Laplanche, *Life and Death in Psychoanalysis*, p. 72.

33 Simon Critchley, *The Problem with Levinas* (Oxford University Press, 2015), pp. 12–13.

34 Critchley, *The Problem with Levinas*, p. 125.

